# A 30-Year Epidemiological Study of Opportunistic Fungal Infections in People Living with HIV in Greece: Associations with Demographic Characteristics and Immune Status

**DOI:** 10.3390/jcm14175936

**Published:** 2025-08-22

**Authors:** Theodora Douvali, Vasilios Paparizos, Varvara Vasalou, Stamatios Gregoriou, Vasiliki Chasapi, Dimitrios Rigopoulos, Alexander J. Stratigos, Electra Nicolaidou

**Affiliations:** 1 Department of Dermatology and Venereology, NHS, Andreas Syggros Hospital of Skin and Venereal Diseases, 161 21 Athens, Greece; tdouvali@gmail.com (T.D.); chasapiresearch@gmail.com (V.C.); 2 1st Department of Dermatology and Venereology, Athens Medical School, National and Kapodistrian University of Athens, “Andreas Syggros” Hospital for Skin and Venereal Diseases, 106 79 Athens, Greece; vpaparizos@yahoo.gr (V.P.); vvasalou@gmail.com (V.V.); dimitrisrigopoulos54@gmail.com (D.R.); alstrat2@gmail.com (A.J.S.); electra.nicol@gmail.com (E.N.)

**Keywords:** opportunistic fungal infections, HIV, PLHIV, CD4+ cell counts, Greece

## Abstract

**Background/Objectives:** Opportunistic fungal infections are common among people living with HIV (PLHIV) and contribute substantially to morbidity, mortality, and hospitalization rates in this population. This study aimed to determine the prevalence of dermatological manifestations of fungal infections in HIV-positive patients and examine their association with demographic, clinical, and immunological characteristics. **Methods:** A retrospective review of medical records from 2500 PLHIV treated at the Infectious Diseases Unit of “Andreas Syggros” Hospital for Skin and Venereal Diseases between 1988 and 2017. Data from patients diagnosed with opportunistic fungal infections were analyzed. Participants were classified as either antiretroviral therapy (ART)-naïve or already receiving treatment. Recorded fungal infections were correlated with epidemiological variables and CD4+ T-cell counts. **Results:** Opportunistic fungal infections were identified in 859 patients (34.36%), with a marked male predominance. Candidiasis was the most frequently reported condition, with a higher prevalence among female patients. Lower CD4+ counts were significantly associated with an increased risk of cryptococcal meningitis, esophageal candidiasis, Pneumocystis jirovecii pneumonia (PJP), and oral candidiasis, whereas higher CD4+ counts were more common in patients with dermatophytosis, onychomycosis, and pityriasis/tinea versicolor. **Conclusions:** Opportunistic fungal infections remain highly prevalent in PLHIV, particularly among those with advanced immunosuppression. CD4+ T-cell counts are key diagnostic and prognostic markers, reinforcing their importance in monitoring disease progression and guiding clinical management.

## 1. Introduction

Human Immunodeficiency Virus (HIV) is a sexually transmitted disease that compromises the immune system and increases susceptibility to life-threatening opportunistic infections, including those of fungal origin. HIV infection remains a major global public health concern, having caused an estimated 44.1 million deaths to date and affecting not only health outcomes but also economic development and social stability [1,2,3,4,5]. According to the World Health Organization (WHO), approximately 40.8 million people were living with HIV in 2024, with 1.3 million new cases reported that year [1].

Greece has experienced a considerable burden of HIV/AIDS, with 20,627 HIV cases reported to the National Public Health Organization (N.P.H.O.) by 31 December 2023, and 2186 deaths among AIDS cases [6]. Of these, 82.1% (16,937) occurred in males, with the predominant age group at diagnosis being 30–39 years, followed by 40–49 years [6]. The most frequently reported mode of HIV transmission is unprotected male-to-male sexual contact, accounting for 47.9% of the diagnosed HIV cases in Greece as of 31 December 2023 [6]. The introduction of highly active antiretroviral therapy (HAART) in 1996 has markedly improved survival rates and quality of life among people living with HIV (PLHIV), reducing AIDS-related mortality and delaying clinical disease progression [7,8,9].

Opportunistic fungal infections are common in PLHIV, due to immunosuppression and have a substantial impact on morbidity, mortality, and hospitalization rates [10,11]. Dermatological disorders such as skin eruptions (e.g., xerosis, acne), opportunistic fungal infections (e.g., herpes simplex, varicella-zoster, candidiasis, dermatophytosis, histoplasmosis, cryptococcosis, Pneumocystis), AIDS-related malignancies (e.g., Kaposi’s sarcoma), and antiretroviral therapy (ART)-associated drug eruptions are among the most frequent clinical manifestations of HIV/AIDS [12]. Some of these, such as oral-esophageal Candidiasis and Pneumocystis jirovecii pneumonia, may also serve as markers of HIV disease progression [12].

Epidemiological data on the frequency and distribution of fungal infections in Greece—both in the general population and among PLHIV—remain extremely limited. Nevertheless, environmental and socio-political factors appear to influence fungal epidemiology in the country. Greece’s temperate Mediterranean climate, characterized by mild, humid winters, warm and dry summers, and extended periods of sunlight throughout most of the year, plays a key role in shaping fungal community composition. Higher temperatures and relative humidity, especially during summer, combined with increased sweating and persistent skin moisture, create favorable conditions for the proliferation of fungal pathogens, particularly dermatophytes and yeasts [13].

Furthermore, the financial crisis that affected Greece in late 2008 had a profound negative impact on the management of invasive fungal infections. The significant reduction in public health expenditure led to underdiagnosis and delayed diagnoses, as well as restricted access to antifungal treatments and suboptimal clinical care [14]. These systemic limitations, combined with the country’s geo-climatic variability and regional disparities in healthcare access, may have further shaped the epidemiological patterns of fungal infections during this period.

This study aims to determine the prevalence of dermatological manifestations of fungal infections in a large cohort of PLHIV treated at the Infectious Diseases Unit of “Andreas Syggros” Hospital in Athens over a 30-year period and to examine their associations with demographic and other clinical and immunological parameters.

## 2. Materials and Methods

### 2.1. Study Design and Participants/Subjects

This monocentric, retrospective, descriptive cohort study analyzed the medical records of 2500 PLHIV who received treatment at the Infectious Diseases Unit of “Andreas Syggros” Hospital, a tertiary care referral center for dermatological disorders in Athens, from 1988 to 2017. The Infectious Diseases Unit specializes in diagnosing, monitoring, and treating HIV patients. Data from individuals diagnosed with fungal infections were extracted and analyzed. Participants were either ART-naïve (initiating therapy after being diagnosed at the hospital) or already receiving treatment. ART-naïve patients were defined as those who had not received antiretroviral therapy prior to the diagnosis of a fungal infection, whereas ‘treated’ patients had been on ART for at least 30 days before diagnosis. Treatment regimens varied, encompassing early nucleoside reverse transcriptase inhibitor (NRTI)-based therapies, highly active antiretroviral therapy (HAART), and later integrase inhibitor-based combinations. Immune status was assessed using CD4+ T-cell counts, acknowledging that ART exposure did not consistently result in immune reconstitution, particularly during earlier treatment eras or in individuals presenting late in the course of infection. Data collection was based on medical records and included demographic information (e.g., gender, age), ART history, laboratory results, and clinical outcomes. This study included patients diagnosed with a fungal infection within the first two months of HIV diagnosis.

Various clinical specimens (including oral swabs, sputum, and skin/nail samples) were collected according to the patient’s clinical presentation. Specimens were obtained under aseptic conditions and processed in the microbiology laboratory for fungal infection confirmation. The diagnosis of fungal infections was established through a combination of clinical assessment and laboratory confirmation, including direct microscopic examination, culture, and, where available, histopathological or serological testing. Specimens were subjected to direct microscopy using 10% or 20% potassium hydroxide (KOH) mounts and/or calcofluor white staining. Cultures were performed on a Sabouraud dextrose agar supplemented with chloramphenicol and actidione (cycloheximide), incubated at 25–30 °C. In cases of subcutaneous and systemic mycoses, serological assays were used to detect specific antibodies (e.g., histoplasmosis), and fungal antigens (e.g., cryptococcosis, aspergillosis, candidiasis, histoplasmosis) were identified. Molecular diagnosis using polymerase chain reaction (PCR) was also employed for the diagnosis and confirmation of fungal infections with higher sensitivity.

### 2.2. Assessment of Immune Status

CD4+ cell count was determined for each patient enrolled in our study by flow cytometry using the fluorescent-activated cell sorter BD FACS Count system (Becton Dickinson) following the manufacturer’s instructions. HIV viral RNA quantification was performed by polymerase chain reaction (PCR).

### 2.3. Data Analysis

Data were analyzed using SPSS software (version 28.0.1.1, IBM, Armonk, NY, USA). Categorical variables were expressed as frequencies and percentages, while continuous variables were summarized using means and standard deviations. Chi-square tests and *t*-tests were used to compare categorical and continuous variables, respectively. Statistical significance was set at *p* ≤ 0.05.

## 3. Results

From 1988 to 2017, among the 2500 HIV-positive outpatients treated at the Infectious Diseases Unit of the “Andreas Syggros” Hospital in Athens, 859 (34.36%) presented with fungal infections. Of these 859 patients, 744 were males and 115 were females. A marked gender disparity was observed, with participants being predominantly male (86.6%, n = 744), and a median age of 34 years (range: 15–79 years). Patients aged 30–39 years accounted for the highest proportion (40.74%), followed by those aged 20–29 years (26.9%), 40–49 (18.74%), >50 (12.92%), and <20 (0.7%) years (Table 1). Between 1988 and 1997, 503 HIV patients with fungal infections were treated, while the number of new infections diagnosed significantly decreased during the period 1998–2007, with 230 new cases, and from 2008 to 2017, only 126 new cases were recorded (Table 1).

### 3.1. Clinical Characteristics

Individuals were diagnosed with HIV/AIDS through clinical symptoms or routine screening (Table 2). The most common reasons for diagnosis were as follows: 11.99% (N = 103) due to HIV positivity in spouses or sexual partners, 11.64% (N = 100) were screened at their request, 6.05% (N = 52) presented with a fever, 5.47% (N = 47) after blood donation, 3.96% (N = 34) following a diagnosis of Kaposi’s sarcoma (KS), 3.49% (N = 30) after being diagnosed with PJP (Pneumocystis jirovecii pneumonia), 2.68% (N = 23) due to a lymph nodes diagnosis, 2.56% (N = 22) due to a Candida diagnosis, 2.56% (N = 22) due to a Syphilis diagnosis, and 2.21% (N = 19) after a diagnosis of genital warts. All patients were diagnosed with fungal infections within the first 2 months after their HIV/AIDS diagnosis, through laboratory-based mycological testing.

Furthermore, in the majority of cases, the patients were diagnosed—through a mycological study—with various opportunistic fungal infections, both cutaneous and systemic (Table 3). The patients presented with multiple concomitant infections. Clinical evaluation showed that oral candidiasis (thrush) was the most common and recurrent fungal infection, occurring in 72.6% of the cases, followed by PJP (21.1%), esophageal candidiasis (9.8%), pityriasis/tinea versicolor (9.4%), onychomycosis (primarily due to *Trichophyton rubrum*) (8.7%), plantar epidermophytosis (3.4%), and cryptococcal meningitis (1.0%).

Besides fungal infections, 269 individuals (31.31%) in our cohort also presented with various comorbidities. The most prevalent comorbidities among HIV patients were as follows: depressive disorder (3.95%), duodenal ulcer (2.56%), psychosis/schizophrenia (2.21%), drug addiction (1.98%), allergic rhinitis (1.86%), and psoriasis (1.63%) (Table 4).

Furthermore, to explore the impact of gender on the prevalence of cutaneous and systemic fungal infections in PLHIV, a chi-square test of independence was performed. The results indicate statistically significant differences between males and females in four cases. Gender differences were observed, with males exhibiting a higher prevalence of PJP (χ^2^(1) = 6.35, *p* = 0.012) and pityriasis/tinea versicolor (χ^2^(1) = 5.411 *p* = 0.020), while females showed higher rates of oral candidiasis and tinea capitis (χ^2^(1) = 10.127, *p* = 0.001 and χ^2^(1) = 6.534, *p* = 0.011, respectively) (Table 5).

### 3.2. Impact of HAART Therapy

Following the introduction of HAART in 1996, the incidence of systemic infections (PJP and cryptococcal meningitis) and mucocutaneous infections (oral and esophageal candidiasis) significantly declined (Table 6). Onychomycosis, plantar epidermophytosis, and pityriasis/tinea versicolor showed increased prevalence post-1996 (Table 6). However, dermatophytosis, tinea capitis, cryptococcal meningitis, pulmonary candidiasis, and candidal meningitis demonstrated no statistically significant differences before or after the introduction of HAART (Table 6).

### 3.3. CD4+ Cell Counts and Viral Load Status in Relation to Fungal Infections

CD4+ cell counts were measured within the first two months after both the diagnoses of fungal infections and of HIV/AIDS. Values were available for all patients, ranging from undetectable to 2345 cells/μL. Mean CD4+ cell counts in relation to opportunistic fungal infections, both cutaneous and systemic, are summarized in Table 7. Lower CD4+ counts correlated significantly with oral candidiasis (t = 4.273, *p* = 0.000), PJP (t = 4.572, *p* = 0.000), esophageal candidiasis (t = 3.382, *p* = 0.001), and cryptococcal meningitis (t = 4.799, *p* = 0.001), whereas higher CD4+ counts were associated with dermatophytosis (t = −2.730, *p* = 0.008), pityriasis/tinea versicolor (t = −3.252, *p* = 0.001), and onychomycosis (t = −3.050, *p* = 0.003) (Table 7).

Meanwhile, viral load metrics did not appear to be associated with the prevalence of fungal infections. Viral load status fluctuates significantly depending on the stage of the infection and whether or not treatment is being administered.

## 4. Discussion

Over the past 30 years, HIV/AIDS has remained a major public health challenge in Greece, placing a considerable strain on the Greek healthcare system and contributing to increased morbidity and mortality rates. Although the introduction of HAART has considerably improved the life expectancy and quality of life of PLHIV, reducing AIDS-related deaths and transforming HIV into a manageable chronic condition, the disease remains a public health issue of utmost significance [15]. The AIDS reporting system was initiated in Greece in 1984 (Ministerial Decision A1/6122/19-9-1986) and HIV case reporting started in 1998 (Ministerial Decision Β1/5295/7-8-1998) [6]. For surveillance and trend analysis purposes, HIV infections before 1998 were retrospectively reported to the Hellenic Center for Disease Control and Prevention (HCDCP) in collaboration with the HIV/AIDS Reference Centers and the Infectious Disease Units/Outpatient Clinics for HIV (+) individuals [6].

To date, no population-based epidemiological studies have been conducted in Greece on the incidence of fungal infections. The limited available data derive predominantly from selected high-risk populations, where under-reporting is considered substantial [16]. These findings may also be influenced by Greece’s climate, characterized by mild, wet winters and hot, dry summers, as well as by its geographical diversity [16]. In this context, the distribution of pathogens observed in our cohort could plausibly also reflect the impact of local climatic conditions. The hot and humid periods of a Greek summer create an environment favorable to the proliferation and transmission of dermatophytes, non-dermatophyte molds, and yeasts [17].

Our findings highlight the significant burden of opportunistic fungal infections among PLHIV, emphasizing their correlation with immune status. We found that HIV related opportunistic fungal infections occurred in 859/2500 (34.36%) of the treated patients, a considerably higher rate than in the world’s general population, where it is estimated to be 20–25% [18], with candidiasis predominating. Gender disparity and male predominance (χ^2^(1) = 460.6, *p* < 0.0001) were observed throughout the study period (1988–2017), with 744 (86.6%) affected men and 115 women (13.4%). Globally, the vast majority of new HIV cases occur in men through homosexual transmission [1]. For example, in the United States in 2022, 67% (21,400 of the 31,800) of all new HIV cases were among gay and bisexual men, while only 22% of the infections reported (2100 men and 4900 women) were among people who contracted the virus through heterosexual transmission, and 7% (2300 cases) occurred in individuals who injected drugs [19].

PLHIV, as their disease progresses, are at a higher risk of developing both common and opportunistic fungal infections than the general population, due to their immunosuppressed state. These infections manifest in relation to the severity of CD4+ count suppression [20]. In our study, the main clinical symptoms that led to the diagnosis of patients with HIV/AIDS were fever, Kaposi’s sarcoma, PJP, lymph nodes, candidiasis, syphilis, and genital warts. All participants were diagnosed with fungal infections within the first 2 months after their HIV/AIDS diagnosis, while the most common, recurrent, and concomitant infections among the participants were the following: oral candidiasis (72.6%), PJP (21.1%), esophageal candidiasis (9.8%), pityriasis/tinea versicolor (9.4%), onychomycosis (8.7%), and plantar epidermophytosis (4.1%), which is in line with other analyses [21,22,23].

Male patients showed a higher prevalence of PJP and pityriasis/tinea versicolor, while female patients were more likely to present with oral candidiasis and tinea capitis. This gender disparity may be attributed to differences in immune response, exposure risks, and health-seeking behaviors. Gender-based differences in fungal infection prevalence have been observed in multiple studies. In a USA retrospective cross-sectional study of all hospital admissions with a primary discharge diagnosis of HIV between 2002 and 2014, males had a higher prevalence of PJP compared to females (70% vs. 30%, *p* < 0.0001), where the majority of the patients were Black people, followed by Caucasian and Hispanic people [24]. In another study that aimed to characterize the clinical and immunological impact that PJP and its treatment have in patients with advanced HIV (CD4+ count ≤ 100 cells/µL) who were starting ART, scientists concluded that males were more likely to have been diagnosed with PJP than females (odds ratio [OR], 2.68; 95% CI, 1.34–5.38; *p* = 0.004) [25]. Moreover, researchers in an Ethiopian study assessing the prevalence and associated factors of pityriasis/tinea versicolor among patients attending the Dermatovenereology clinic at the University of Gondar Comprehensive Specialized Hospital Magnitude (for any reason) concluded that the odds of experiencing pityriasis versicolor among the male patients were 4.19 (AOR = 4.19, 95% CI: 1.92, 9.14) times higher than for the female patients [26]. However, another cross-sectional, descriptive study in South Africa, examining the prevalence and spectrum of dermatoses in a sample of 970 individuals with HIV resulted that there is no significant association between skin diseases (infectious or non-infectious dermatoses) and patient demographics (gender and ethnicity) or HIV-disease characteristics (CD4+ cell count, viral load and duration of antiretroviral therapy [27].

Furthermore, our observation of increased oral candidiasis and tinea capitis prevalence among females is in accordance with Nigerian and Indian studies. Awoyeni et al. (2017) [28] investigated 154 HIV patients in Nigeria and detected Candida species in 39.5% of the patients. The mean age of individuals with HIV with candidiasis was 40.4 years, with the age group of 29–39 years being the most affected (49.2%). The prevalence of candidiasis was significantly higher in females (55, 90.2%) compared to males (6, 9.7%) (*p* = 0.042) [26]. Similarly, Lar et al. (2012) [29] have also demonstrated a higher carriage rate of candidiasis in women (7.26%) than in men (2.41%). On the other hand, Suryana et al. (2020) [30], in a case control study conducted at Wangaya hospital in Indonesia from 1 March 2016 to 30 July 2019, which included 448 participants (207 HIV patients and 241 controls), showed that oral candidiasis, in addition to acting as an early marker for people living with HIV/AIDS, is associated with the male sex (*p* = 0.002; OR = 1.88; 95% CI: 1.26–2.80). In addition, Rao et al. (2012) [31] also found that male individuals with HIV in Southern India had a higher risk of oral lesions, especially oral candidiasis, than females (18.8% males, 10.3% females, *p* = 0.00). Although our observation of an increased prevalence of oral candidiasis among females aligns with findings from studies conducted in Nigeria and parts of India [28,29], this gender-related pattern is not universally observed [30,31]. These conflicting results could reflect a complex interaction of biological, behavioral, and sociocultural factors. For instance, regional differences between men and women in healthcare-seeking behavior might influence the likelihood of diagnosis (e.g., women in certain regions may be more likely to seek or access healthcare services). Additionally, coexisting factors, including comorbidities, nutritional status, and oral hygiene practices, that vary by gender and geographic region may also contribute to these differences. Moreover, methodological differences, such as variations in study design, sample size, diagnostic criteria, and population characteristics, could also lead to the observed discrepancies in research findings.

Our findings, in which female patients showed higher rates of tinea capitis, are consistent with the literature, where gender disparity in the prevalence of tinea capitis has been noted, with higher rates reported among females [32]. Several hypotheses may account for this observation. Postmenopausal women may be particularly susceptible, possibly due to reduced sebum production associated with declining estrogen levels, which results in diminished fatty acid secretion and the loss of scalp acidity [32]. Another explanation for the disproportionate representation of this infection among African American women includes their role as primary caretakers of children with tinea capitis and specific hair care practices—such as less frequent shampooing and traction hair styling—that may facilitate fungal penetration of the hair and scalp [32]. Poor hygiene also continues to contribute to the development of tinea capitis in modern settings. Risk factors further include situations involving close contact with infected individuals or contaminated belongings, such as crowded living environments (e.g., group homes, army barracks), exposure to infected children, participation in contact sports, and the use of shared gym or swimming pool facilities [32]. In addition, close proximity to animals constitutes another potential source of infection, as both domestic pets (cats, dogs) and livestock (e.g., cattle) can serve as reservoirs and transmitters of tinea capitis to humans [32]. Moreover, adherence to HAART may modify infection patterns by affecting overall immune reconstitution, potentially shifting the burden from systemic opportunistic infections toward superficial mycoses in patients with better adherence.

Infectious microbial agents (bacteria, fungi, parasites, or viruses) causing opportunistic fungal infections may be asymptomatic or symptomatic in healthy individuals and are usually self-limiting. However, in immunocompromised individuals and individuals with malignancy, these infections can result in a severe, life-threatening disease [33]. The introduction of HAART therapy has significantly altered the epidemiology of opportunistic fungal infections in PLHIV. While systemic infections such as PJP and cryptococcal meningitis have declined [11,34], superficial infections like onychomycosis and pityriasis/tinea versicolor have shown an increase, possibly due to prolonged survival and immune reconstitution. Similar trends have also been reported in South African and Brazilian cohorts [5,27]. Before 1996, HIV/AIDS was associated with high mortality and significantly reduced life expectancy, particularly in countries with under-resourced healthcare systems. The limited survival time often meant that many individuals succumbed to AIDS-related systemic fungal infections (e.g., PJP, cryptococcal meningitis) before superficial infections like onychomycosis could clinically manifest or progress and be diagnosed [35]. However, the introduction of HAART therapy in 1996 marked a pivotal turning point in the management of HIV infection. This combination therapy effectively suppresses viral replication, leading to substantial improvements in immune function. Consequently, PLHIV have experienced a significantly prolonged life expectancy and improved quality of life [35]. This increase in survival rate has probably led to a higher prevalence and detection of chronic conditions, including superficial fungal infections such as onychomycosis, which now represent a more prominent clinical concern in the HIV-positive population.

CD4+ count and viral load are two critical markers used in the management of HIV patients to assess the health of their immune system and the effectiveness of antiretroviral therapy. CD4+ counts provide information on the overall immune function of an individual with HIV; lower counts indicate increased susceptibility to opportunistic fungal infections. A CD4+ count below 200 cells/μL has been well documented in numerous studies as a significant risk factor for developing infections such as orofacial manifestations (with oral candidiasis being the most common condition), tuberculosis, PJP, and cryptococcal meningitis [36,37,38]. In accordance with the previous studies, we found that PLHIV with lower CD4+ cell counts were more likely to be diagnosed with cryptococcal meningitis (t = 4.799, *p* = 0.001), esophageal candidiasis (t = 3.382, *p* = 0.001), PJP (t = 4.572, *p* = 0.000), and oral candidiasis (t = 4.273, *p* = 0.000). Conversely, our findings suggest that higher CD4+ cell counts (>400 cells/μL) were associated with dermatophytosis (t = −2.730, *p* = 0.008), onychomycosis (t = −3.050, *p* = 0.003), and Pityriasis/Tinea versicolor (t = −3.252, *p* = 0.001). Similarly, Khat et al. (2020) reported a statistically significant association between elevated CD4+ counts (>400 cells/μL) and the presence of dermatophytosis and pityriasis versicolor [39].

Viral load reflects the level of active viral replication. During the early years of the epidemic (1988–1996), before the widespread availability of HAART, viral load was not routinely measured, and CD4+ T-cell count served as the primary marker of immune suppression [40]. After the introduction of HAART in 1996, viral replication could be effectively suppressed in many individuals; however, cellular immunity often remained impaired, particularly in those who initiated treatment late in the course of an infection [35,41]. As a result, fungal infections continued to occur even in patients with low or undetectable viral loads [42]. Opportunistic fungal infections are more directly associated with cellular immune dysfunction—especially CD4+ T-cell counts below 200 cells/μL—than with viral load itself [42]. In this context, patients may exhibit well-controlled viral loads due to HAART but remain immunocompromised and susceptible to fungal disease, particularly if immune reconstitution has not yet occurred or if HAART initiation was delayed [42]. This immunological pattern is reflected in our cohort as well. The viral load metrics in our study did not show a strong correlation with fungal infections in our cohort, suggesting that immune suppression, rather than viral replication, plays a primary role in the development of opportunistic fungal infections. This finding is consistent with several other studies such as those of Lu et al. (2012), who demonstrated low CD counts (<200 cells/μL) and elevated HIV viral loads (from 500 to 5.28 × 107 copies/mL) in 50 AIDS patients with PJP [43], and Williams et al. (1999) who reported that individuals with lower median CD4 cell counts and higher median RNA copies were associated with a significantly higher risk of developing specific opportunistic fungal infections (PJP, CMV and MAC) [44].

Overall, our study reinforces the importance of CD4+ monitoring, as lower counts were strongly associated with severe opportunistic fungal infections. However, HIV viral loads did not show a clear correlation with fungal infection prevalence, suggesting that immune suppression rather than viral replication plays a crucial role in opportunistic fungal disease development. Future studies should explore targeted antifungal prophylaxis strategies and optimize ART regimens to further reduce fungal infection rates and improve patient outcomes [45,46,47].

This study has several limitations inherent to its design. First, it is a 30-year epidemiological, observational, and retrospective study, which introduces constraints related to data availability and consistency over time. Notably, due to the extended study period and the retrospective nature of data collection, a systematic recording of viral loads in Greece was not consistently performed throughout the entire timeframe. Routine viral load monitoring only became widely implemented after the mid-1990s, following the introduction of HAART. Furthermore, in many cases, there may have been a temporal discrepancy between the measurement of viral load and the onset of the fungal infection, which limits the accuracy of the observed associations. This time lag could potentially lead to an underestimation or overestimation of actual viro-immunological status at the time of infection, thereby reducing the accuracy and precision of the correlation analyses. Another key limitation is the variability in the timing of the CD4+ T-cell count measurements in relation to fungal disease onset. While most measurements were obtained within approximately two months of either HIV/AIDS or fungal infection diagnosis, the lack of a strictly standardized schedule means that CD4+ levels may still have varied due to treatment response or disease progression, potentially leading to a misclassification of patients’ immune statuses.

Therefore, the associations identified in this study, though statistically significant, should be interpreted with caution. Future prospective studies with temporally aligned clinical and laboratory data are essential to improve the accuracy of viro-immunological correlations and to clarify the causal relationships in PLHIV.

## 5. Conclusions

HIV-related opportunistic fungal infections were observed in 34.36% of the treated patients in our cohort, with male predominance. Candidiasis was the most commonly reported manifestation, and was highly prevalent among women. Immune status was closely related to several fungal infections. Cryptococcal meningitis, esophageal candidiasis, oral candidiasis, and PJP predominantly occurred in individuals with CD4+ counts below 200 cells/mm^3^, while increased CD4+ levels were found in individuals with dermatophytosis, onychomycosis, and Pityriasis/Tinea versicolor. Moreover, viral load metrics did not demonstrate any significant association between HIV viral loads and fungal infection, but there is a strong correlation depending on the stage of the opportunistic fungal infection and drug administration.

Hence, fungal infections remain a major concern in PLHIV, particularly among those with low CD4+ counts. CD4+ cell monitoring is crucial for predicting infection risk and guiding clinical management. Given the high prevalence and clinical impact of opportunistic fungal infections, timely diagnosis and early intervention are essential in improving patient outcomes.

Building upon these findings, several actionable insights emerge for clinical practice. Monitoring CD4+ T-cell counts remains a critical tool for identifying individuals at elevated risk for opportunistic fungal infections and for determining the need for prophylactic or preemptive antifungal therapy. Clinicians should maintain a high index of suspicion for deep fungal infections in patients with CD4+ counts < 200 cells/mm^3^, regardless of viral load status. Conversely, superficial fungal infections, often observed in patients with higher CD4+ counts, may serve as early indicators of incomplete immune reconstitution or delayed HAART initiation. Implementing routine fungal screening, particularly in patients with persistent immunosuppression, and tailored antifungal management based on immunological profiles can significantly enhance patient outcomes and reduce the burden of HIV-associated fungal infections.

Notably, our findings provide novel insights into the burden and profile of fungal infections in Greece and the broader Mediterranean region—a setting where climatic conditions, healthcare access, and economic constraints distinctively influence HIV care delivery. Effective local antifungal stewardship programs that take into account regional epidemiology, resource availability, resistance patterns, and the misuse of over-the-counter antifungal drugs are essential to optimize treatment outcomes. Future efforts should aim to adapt fungal diagnostic protocols and preventive strategies to the specific needs of Mediterranean PLHIV, with an emphasis on sustainable and equitable healthcare delivery.

Further research is needed to explore innovative antifungal treatments, resistance patterns, and the potential benefits of integrating fungal infection screening into routine HIV care protocols. Enhancing awareness and education on fungal complications among healthcare providers and patients alike will play a vital role in improving long-term disease management and quality of life for PLHIV.

## Figures and Tables

**Table 1:** Demographic characteristics of HIV/AIDS patients with fungal infections.

Characteristics	Category	No. of Cases
Gender	Male	744 (86.6%)
	Female	115 (13.4%)
Age	<20 y	6 (0.7%)
	20–29 y	231 (26.9%)
	30–39 y	350 (40.74%)
	40–49 y	161 (18.74%)
	>50 y	111 (12.92%)
Calendar years	1988–1997	503 (58.5%)
	1998–2007	230 (26.8%)
	2008–2017	126 (14.7%)

**Table 2:** Reasons that lead to HIV/AIDS diagnosis.

	N	%
HIV infected sexual partners	103	11.99%
On their initiative	100	11.64%
Fever	52	6.05%
Blood donation	47	5.47%
Kaposi’s sarcoma (KS)	34	3.96%
PJP (Pneumocystis jirovecii pneumonia)	30	3.49%
Lymph nodes	23	2.68%
Candida	22	2.56%
Syphilis	22	2.56%
Genital warts	19	2.21%
VZV (Varicella Zoster Virus)	13	1.51%
Pulmonary infections	12	1.40%
Diarrhea	11	1.28%
Pre-operational check	11	1.28%
Imprisonment	11	1.28%
Pregnancy	11	1.28%
Weight loss	8	0.93%
Fatigue	8	0.93%
HSV	8	0.93%
TBC	8	0.93%
Neurological complications	6	0.70%
Check up	6	0.70%
Atypical rash	5	0.58%
Dermatological manifestations	5	0.58%
Folliculitis	5	0.58%
Psoriasis	5	0.58%
Other	99	11.52%

**Table 3:** Cutaneous and systemic fungal infections in PLHIV.

Types of Fungal Infections	Cases	%
Cutaneous Fungal Infections		
Oral Candidiasis (Thrush)	624	72.6%
Pityriasis/Tinea Versicolor	81	9.4%
Onychomycosis (due to Trichophyton rubrum)	75	8.7%
Plantar Epidermophytosis	35	4.1%
Tinea Capitis (due to Microsporum canis)	1	0.1%
Systemic Fungal Infections		
PJP	181	21.1%
Esophageal Candidiasis	84	9.8%
Cryptococcal Meningitis	9	1.0%
Pulmonary Candidiasis	1	0.1%
Candidal Meningitis	1	0.1%

**Table 4:** Comorbidities in PLHIV.

Comorbidities	Cases	%
Depressive disorder	34	3.95%
Duodenal ulcer	22	2.56%
Psychosis/Schizophrenia	19	2.21%
Drug addiction	17	1.98%
Allergic rhinitis	16	1.86%
Psoriasis	14	1.63%
Alcoholism	13	1.51%
Nephrolithiasis	12	1.40%
Diabetes	12	1.40%
Hypertension	10	1.16%
Asthma	10	1.16%
Other	90	10.48%

**Table 5:** Cutaneous and systemic fungal infections in PLHIV by gender.

	Males				Females				χ^2^	*p*
	No		Yes		No		Yes			
	N	%	N	%	N	%	N	%		
Cutaneous Fungal Infections										
Oral Candidiasis (Thrush)	217	29.2	527	70.8	17	14.9	97	85.1	10.127	0.001
Pityriasis/Tinea Versicolor	667	89.7	77	10.3	110	96.5	4	3.5	5.411	0.020
Onychomycosis	676	90.9	68	9.1	107	93.9	7	6.1	1.115	0.291
Plantar Epidermophytosis	711	95.6	33	4.4	112	98.2	2	1.8	1.816	0.178
Tinea Capitis	744	100	0	0	113	99.1	1	0.9	6.534	0.011
Systemic Fungal Infections										
PJP	576	77.5	167	22.5	101	87.8	14	12.2	6.350	0.012
Esophageal Candidiasis	668	89.8	76	10.2	106	93.0	8	7.0	1.144	0.285
Cryptococcal Meningitis	735	98.8	9	1.2	115	100	0	0	1.406	0.236
Pulmonary Candidiasis	743	99.9	1	0.1	115	100	0	0	0.155	0.694
Candidal Meningitis	742	99.9	1	0.1	115	100	0	0	0.155	0.694

**Table 6:** Occurrence of cutaneous and systemic fungal infections during the periods 1988–1995 and 1996–2017.

Opportunistic Fungal Infections	Opportunistic Fungal Infections 1988–1995		Opportunistic Fungal Infections 1996–2017		χ^2^	*p*
	ν	%	ν	%		
**Cutaneous fungal infections**						
Oral Candidiasis (thrush)	331	80.15	292	65.47	23.174	<0.00001
Pityriasis/Tinea Versicolor	20	4.84	61	13.68	19.5965	<0.00001
Onychomycosis	22	5.53	53	11.98	11.5678	0.000671
Dermatophytosis	26	6.30	49	10.99	1.4833	0.223263
Plantar Epidermophytosis	7	1.69	28	6.28	11.524	0.000687
Tinea Capitis	1	0.24	0	0	1.084	0.298
**Systemic fungal infections**						
PJP	131	32.11	50	11.21	54.229	<0.00001
Esophageal Candidiasis	56	13.56	28	6.28	12.8859	0.000331
Cryptococcal Meningitis	6	1.45	3	0.67	1.2588	0.261876
Pulmonary Candidiasis	1	0.24	0	0	1.084	0.298
Candidal Meningitis	0	0	1	0.22	0.924	0.337

**Table 7:** CD4+ dynamics in cutaneous and systemic fungal infections in HIV/AIDS patients.

Opportunistic Fungal Infections	No		Yes		t	*p*-Value
	Mean	SD	Mean	SD		
**Cutaneous fungal infections**						
Oral Candidiasis (thrush)	390.6	370.5	275.8	288.6	4.273	0.000
Pityriasis/Tinea Versicolor	296.0	314.7	415.8	321.7	−3.252	0.001
Onychomycosis	297.3	316.3	411.7	309.6	−3.050	0.003
Dermatophytosis	293.7	292.8	449.2	485.0	−2.730	0.008
Plantar Epidermophytosis	304.4	316.5	390.6	330.5	−1.381	0.177
Tinea Capitis	307.7	317.2	4.0	NA	NA	NA
**Systemic fungal infections**						
PJP	329.6	324.0	220.2	272.6	4.572	0.000
Esophageal Candidiasis	315.9	323.7	220.8	232.9	3.382	0.001
Cryptococcal Meningitis	308.9	317.8	111.9	118.7	4.799	0.001
Pulmonary Candidiasis	307.1	317.1	37.0	NA	NA	NA
Candidal Meningitis	307.1	317.1	46.0	NA	NA	NA

Note: NA denotes not available. CDC stages of HIV infection: Stage 1 (>500 cells/μL), Stage 2 (200–499 cells/μL), and Stage 3 (<200 cells/μL).

## Data Availability

Data are available on request due to restrictions related to patient confidentiality, as the data include medical history information and are subject to the protection of sensitive personal data.

## Abbreviations

Human Immunodeficiency Virus (HIV); World Health Organization (WHO); National Public Health Organization (N.P.H.O.); highly active antiretroviral therapy (HAART); people living with HIV (PLHIV); Pneumocystis jirovecii pneumonia (PJP).
